# Supplementary material for: A Study on the Behavior Patterns of Liquid Aerosols Using Disinfectant Chloromethylisothiazolinone/Methylisothiazolinone Solution
Source: Molecules. 2021 Sep 22;26(19):5725. doi: 10.3390/molecules26195725 (PMC8510451; doi:10.3390/molecules26195725)
Supplement: Supplementary file 1 [file molecules-26-05725-s001.zip › molecules-1350802-supplementary.pdf]

molecules

Supplementary Materials

A Study on the Behavior Patterns of Liquid Aerosols Using Disinfectant Chloromethylisothiazolinone/ Methylisothiazolinone Solution

Yong-Hyun Kim [1,*,†], Mi-Kyung Song [2,3,4,†] and Kyuhong Lee [2,3,4,*]

1 Department of Environmental Environment, Sangji University, Wonju 26339, Korea
2 Inhalation Toxicology Center for Airborne Risk Factor, Korea Institute of Toxicology, Jeongeup 56212, Korea; mikyung.song@kitox.re.kr
3 Humidifier Disinfectant Health Center, Korea Institute of Toxicology, Jeongeup 56212, Korea
4 Human and Environmental Toxicology, University of Science & Technology (UST), Daejeon 34113, Korea
* Correspondence: yhkim2021@sangji.ac.kr (Y.-H.K.); khlee@kitox.re.kr (K.L.); Tel.: +82-33-730-0442 (Y.-H.K.); +82-63-570-0881 (K.L.)
† These authors equally contributed to this work.

Table S1. Preparation of the working standards and sample solutions.

A. Working standards for the calibration experiments

Order	Compound	A. Primary standard	B. Working standard (WS): Concentration (ng μL^{-1})							
			Method A				Method B			
		Concentration (%)	WS-1	WS-2	WS-3	WS-4	WS-5	WS-6	WS-7	WS-8
1	MIT	0.43	47.8	120	239	478	431	1,148	2,153	4,306
2	CMIT	1.09	121	303	606	1,213	1,092	2,911	5,458	10,916
Mixing recipe: in volume (μL) [1]:		Primary standard [2]	20	50	100	200	180	480	900	1,800
		Distilled water	1,780	1,750	1,700	1,600	1,620	1,320	900	0
		Total	1,800	1,800	1,800	1,800	1,800	1,800	1,800	1,800

B. Sample solutions for the generation of the CMIT/MIT aerosols

Order	Compound	A. Primary solution	B. Sample solution (SS): Concentration (ng μL^{-1})		
		Concentration (%)	SS-1	SS-2	SS-3
1	MIT	0.368	23.8	47.7	71.5
2	CMIT	1.124	72.8	146	218
Mixing recipe: in volume (mL) [1]:		Primary solution [3]	2.5	5	8
		Distilled water	497.5	495	493
		Total	500	500	500

[1] Mixing conditions and formula for primary standards, primary solution, working standard (WS), and sample solution (SS) are given. [2] Primary standard (Product no.: PHR1597) was purchased from Sigma-Aldrich (USA); Concentrations = 0.43% (MIT) and 1.09% (CMIT) with H_2O. [3] Primary solution (Product no.: 00010039070) was purchased from Dow chemical Group company; Concentrations = 0.368% (MIT) and 1.124% (CMIT) with H_2O.
